# Artificial Intelligence for the Early Detection of Patients with Cognitive Impairment: A Scoping Review

**DOI:** 10.3390/healthcare14060768

**Published:** 2026-03-18

**Authors:** María Moreno-Pineda, Víctor Ortiz-Mallasén, Águeda Cervera-Gasch

**Affiliations:** 1 Faculty of Health Sciences, Universitat Jaume I, Avinguda de Vicent Sos Baynat, s/n, 12071 Castellón de la Plana, Spain; mmorenop372@gmail.com; 2 Hospital Vithas Castellón, Calle Santa María Rosa Molas, 25, 12004 Castellón de la Plana, Spain; 3 Nursing Research Group (GIENF Code 241), Nursing Department, Universitat Jaume I, Avinguda de Vicent Sos Baynat, s/n, 12071 Castellón de la Plana, Spain; cerveraa@uji.es

**Keywords:** artificial intelligence, early diagnosis, cognitive dysfunction, ethics, machine learning, dementia

## Abstract

**Highlights:**

**What are the main findings?**

- Artificial Intelligence tools can contribute to the early detection of subtle cognitive changes, which may be challenging for healthcare professionals to identify.
- The review included 14 studies, mainly systematic reviews and diagnostic studies, supporting the clinical utility of Artificial Intelligence-based approaches.

**What are the implications of the main findings?**

- Artificial Intelligence-based tools could improve clinical decision-making and early intervention strategies in cognitive impairment.
- The use of Artificial Intelligence raises ethical considerations, particularly concerning patient privacy and data security.

**Abstract:**

**Background/Objectives:** Cognitive impairment affects multiple brain functions, and its early detection is essential to prevent progression to dementia; artificial intelligence has shown considerable potential in this field. This scoping review aims to map the impact of artificial intelligence–based tools for the early detection of cognitive impairment by identifying the main technologies used, examining their effectiveness, and exploring their ethical implications. **Methods:** A scoping review was conducted between April and May 2025 following the PRISMA-ScR methodological framework; the review protocol was previously registered on the Open Science Framework. PubMed, Scopus, and Cochrane databases were searched using natural language and controlled vocabulary terms via Medical Subject Headings. The search was limited to articles published between 2020 and 2025, in English or Spanish, with free full-text access. Methodological quality was assessed using CASPe, JBI, and MMAT. **Results:** A total of 14 studies were included after the selection and critical appraisal process. The findings show that artificial intelligence–based tools such as deep-learning models applied to neuroimaging, speech and gait analysis, electronic health record analysis, and mobile health applications demonstrate promising accuracy in detecting early cognitive changes. These technologies enable the identification of subtle patterns that may be difficult to detect using conventional clinical assessments. **Conclusions:** AI-based tools can provide substantial support for clinical decision-making by effectively identifying subtle changes that are imperceptible to human intelligence. However, their use also raises ethical issues related to patient privacy and data security.

## 1. Introduction

Cognitive impairment is a multifactorial condition that affects higher brain functions, including memory, thinking, learning, language, attention, judgment, and decision-making. It represents an intermediate stage between normal cognitive aging and dementia [1,2]. These higher functions enable individuals to perform daily activities effectively. When altered by conditions such as cognitive impairment, they may lead to difficulties ranging from planning activities or solving complex problems to performing simple tasks [3].

The main risk factors include age (especially over 65 years), sex (more frequent in women), marital status (more common among separated or divorced individuals), educational level (often in individuals with no formal education), and living environment (particularly those residing in long-term care facilities) [4]. Chronic conditions such as diabetes, obesity, and hypertension, as well as lifestyle factors including smoking and reduced physical, mental, and social activity, have been associated with an increased risk of cognitive impairment [5].

As advanced age is the primary risk factor, increasing life expectancy has led to a rise in incidence, making cognitive impairment a global health problem affecting millions of people [6]. This condition has a significant overall impact, affecting biopsychosocial health and, consequently, the economic sphere with associated costs estimated at approximately EUR 30.000 per person per year according to 2019 data. Early detection of cognitive alterations before progression to more severe stages is therefore essential. Timely identification allows preventive measures, such as cognitive stimulation through mental exercises, to slow progression toward more severe conditions such as dementia [6,7].

Currently, cognitive impairment affects approximately 50 million people worldwide. Projections suggest that by 2050, these figures will reach 150 million globally and nearly 1 million nationally in Spain if preventive measures supported by early diagnosis are not implemented [8].

It is estimated that 10–15% of cases progress to dementia in subsequent years, with 8–15% developing dementia immediately [2,4].

Early detection remains a challenge for healthcare professionals, mainly because of the absence of clear symptoms in the early stages and the limited sensitivity of conventional methods, which focus exclusively on cognitive assessment. Although useful, these tools often fail to detect subtle changes because early cognitive impairment is progressive and fluctuating. In this context, and given the recent expansion of artificial intelligence (AI) in healthcare, innovative research has emerged seeking to integrate this technology into the early detection of cognitive impairment [9].

AI refers to technology designed to develop computer programs capable of performing tasks similar to those carried out by the human mind [10]. This is achieved through algorithms that process large-scale data and make decisions based on patterns learned through machine learning, enabling systems to learn autonomously without explicit programming [11].

From a theoretical perspective, the integration of AI into healthcare is closely linked to the development of data-driven medicine, which enables the analysis of large and complex datasets to identify patterns associated with disease onset and progression. In the context of cognitive impairment, AI-based approaches can integrate multimodal data sources, including neuroimaging, behavioral indicators, speech patterns, and electronic health records, allowing the identification of subtle changes that may precede clinical diagnosis. These capabilities have positioned AI as a promising tool for early detection and to enhance clinical decision-making in neurodegenerative conditions [12].

In healthcare, AI can play a transformative role. Nurses are often the first professionals to detect early signs of cognitive impairment due to their continuous and direct patient contact. In this context, AI can improve the quality of care by optimizing data collection and analysis, personalizing treatments, monitoring patients, prioritizing tasks, and automating documentation, among other aspects [12,13]. AI, therefore, represents a promising support for nursing practice by facilitating more precise analysis of large volumes of clinical data [10,12].

This study aims to analyze the extent to which AI can enable earlier and more accurate detection compared with traditional tools and whether its practical application contributes to improved patient outcomes. Effectiveness in real-world settings and ethical implications are examined to ensure safe and efficient integration into healthcare systems.

Although several reviews have explored the use of AI for dementia and cognitive impairment detection, many focus primarily on algorithmic performance or specific technological approaches. In contrast, this scoping review aims to provide a broader overview by mapping the different AI-based tools currently used for the early detection of cognitive impairment, while also examining their clinical applicability and associated ethical implications. This perspective is particularly relevant for healthcare professionals involved in the early identification of cognitive decline, such as nurses, who often play a key role in the continuous monitoring and early recognition of cognitive changes in patients.

Accordingly, the objective of this scoping review was to map the impact of AI-based tools on the early detection of cognitive impairment, identify these tools, and explore their effectiveness and ethical implications.

## 2. Materials and Methods

### 2.1. Design

A scoping review was conducted between April and May 2025 following the PRISMA Extension for Scoping Reviews (PRISMA-ScR) framework [14] to identify, select, examine, and synthesize studies addressing AI-based tools for early detection of cognitive impairment. Prior to conducting the review, a protocol was developed and registered in March 2025 on the Open Science Framework (https://doi.org/10.17605/OSF.IO/2RKTS accessed on 14 March 2026).

Based on the intention to map this research field, a research question was formulated using the PCC framework recommended by the Joanna Briggs Institute (JBI) [15] for scoping reviews: Population (P): individuals with early symptoms of cognitive impairment without a definitive diagnosis; Concept (C): AI-based tools; Context (C): early detection of cognitive impairment. Accordingly, the main research question of this scoping review was: How does the use of AI influence the early detection of patients with cognitive impairment?

### 2.2. Eligibility Criteria

Only studies focused primarily on the application of AI for the early detection of cognitive impairment were selected. Following the methodological recommendations of Peters et al. [16] for the development of a scoping review, studies with diverse designs were considered, including systematic reviews, randomized controlled trials, observational studies, and pilot studies. All studies had to focus exclusively on older adult populations. Compliance with predefined inclusion and exclusion criteria was subsequently verified (Table 1).

The eligibility criteria were defined to identify studies that specifically address the application of AI-based approaches for the early detection of cognitive impairment. Studies were eligible if they examined AI tools designed to support screening, prediction, or early diagnostic processes for cognitive decline in older adults. Studies focusing exclusively on advanced stages of dementia, unrelated technological applications, or populations not relevant to the research question were excluded.

The search was limited to studies published between 2020 and 2025 to capture the most recent evidence available at the time the review was conducted. As the review was performed in 2025, this timeframe was selected to include studies published within the previous five years, focusing on the most recent developments in AI technologies and their applications in the early detection of cognitive impairment.

### 2.3. Information Sources

To comprehensively identify relevant studies addressing the objectives of this scoping review, a systematic search was conducted in three internationally recognized health sciences databases: PubMed, Scopus, and Cochrane Library. These sources were selected for their broad coverage of biomedical and health sciences literature, scientific rigor, academic recognition, and continuous updating of the literature.

### 2.4. Search Strategy

To design a sensitive search strategy in line with PRISMA-ScR guidelines [14], broad thematic terms were used rather than relying exclusively on controlled vocabulary. Descriptor selection was based on the clinical experience of two members of the research team (M.M.-P., V.O.-M.), who identified key terms from the PCC-structured clinical question.

Natural language terms were translated into controlled English terms using Medical Subject Headings (MeSH) [17]. Terms were combined using the Boolean operators “AND” and “OR”. A consensus search strategy consisting of three searches was established (Table 2) and applied across all databases, with necessary adaptations while maintaining consistency.

### 2.5. Study Selection

The screening process was conducted independently by two researchers, who reviewed titles, abstracts, and full-text articles according to the predefined eligibility criteria. Disagreements were resolved through discussion until a consensus was reached. Finally, the methodological quality of the remaining studies was evaluated.

### 2.6. Data Extraction Process

Data were extracted using a standardized form including general study information (authors, year), sample characteristics (size, age), intervention description (content, duration), control description, measurement instruments, and primary outcomes.

### 2.7. Methodological Quality Assessment

To ensure an appropriate methodological quality assessment, critical appraisal tools were selected based on the design of the included studies. The Spanish version of the Critical Appraisal Skills Programme (CASPe) [18,19] was used to evaluate systematic reviews, scoping reviews, diagnostic studies, and literature reviews. The Joanna Briggs Institute (JBI) [15] critical appraisal tools were applied to conceptual reviews, cross-sectional studies, and study protocols. Finally, the Mixed Methods Appraisal Tool (MMAT) [20,21] was used to appraise the qualitative studies. This approach allowed each study to be assessed using a tool specifically designed for its methodological design.

Although the appraisal tools used do not establish a fixed cutoff score, studies scoring 4 or higher were considered to have moderate to high methodological quality, as this threshold indicates that the majority of methodological criteria were met. This approach is consistent with methodological guidance suggesting that quality appraisal tools should be used to support transparent evaluation rather than as rigid exclusion criteria [22].

The methodological quality of the included studies was independently assessed by two researchers using the selected appraisal tools. Discrepancies between reviewers were discussed and resolved by consensus.

### 2.8. Results Synthesis

Given the heterogeneity of included studies, findings were synthesized narratively. To this end, results were grouped according to methodological characteristics. Likewise, subcategories were established for primary and secondary outcomes of interest. Comparative analysis explored similarities and differences between significant and non-significant findings, considering interventions, population characteristics, and other methodological aspects.

## 3. Results

### 3.1. Study Selection

A total of 2945 records were initially identified: 2427 (82.41%) from PubMed, 262 (8.9%) from Scopus, and 256 (8.69%) from Cochrane. The higher proportion of records retrieved from PubMed reflects its extensive coverage of biomedical and clinical research related to cognitive impairment and AI applications in healthcare.

**Figure 1:**
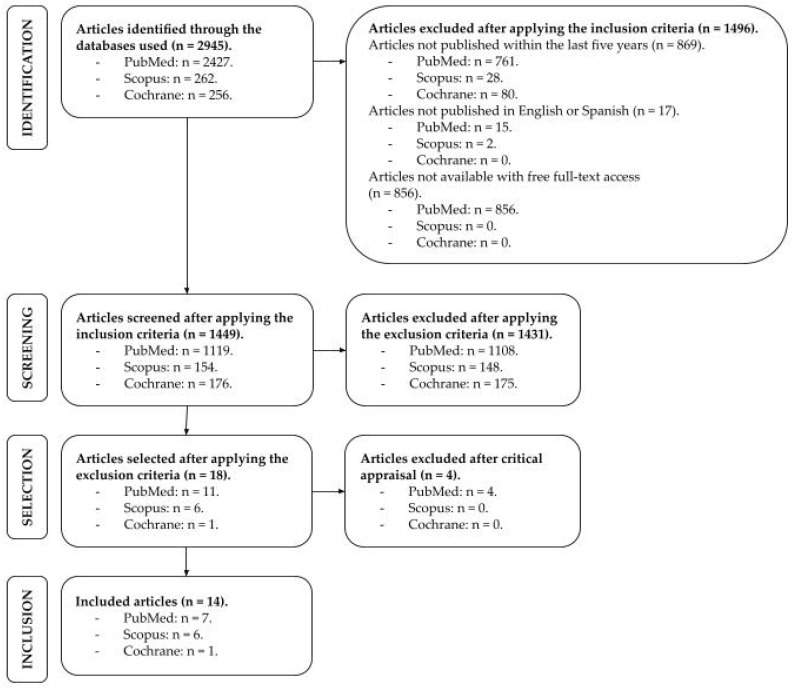
Flowchart of the Articles Selection Process.

After applying the inclusion criteria, 1496 articles were excluded, leaving 1499 records. Upon applying the exclusion criteria, 1431 articles were excluded, reducing the sample to 18 articles. After methodological quality assessment, 4 articles were excluded, resulting in 14 studies included in the final review (Figure 1).

Despite the broad initial search strategy, the number of included studies was reduced after applying the predefined eligibility criteria and methodological quality assessment. The main reasons for exclusion were studies that did not specifically address the use of AI for early detection of cognitive impairment, studies that focused on advanced stages of dementia, and articles that did not meet the predefined eligibility criteria for this review.

### 3.2. Methodological Quality Assessment

**Figure 2:**
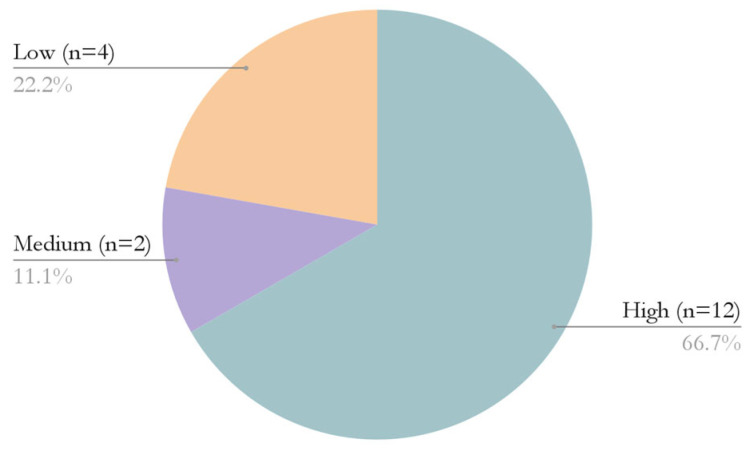
Classification of Articles According to Methodological Quality.

In this scoping review, the methodological quality of the articles was assessed using different checklists for each study design, assigning 1 point to affirmative responses, 0.5 to uncertain responses, and 0 to negative responses. Scores were standardized on a 10-point scale to ensure uniformity. Methodological quality was classified as high for scores equal to or greater than 7 (n = 12; 66.67%), moderate for scores between 4 and 6.9 (n = 2; 11.11%), and low for scores below 4 (n = 4; 22.22%) (Figure 2). Only studies with moderate to high methodological quality were included in the review, as established in the selection criteria.

### 3.3. Study Characteristics

Among the 14 included studies, diagnostic studies predominated (n = 3; 21.44%), although a wide variety of designs were identified. Most studies demonstrated high methodological quality (n = 12; 85.71%) and were published in 2020 (n = 5; 35.75%). Half were retrieved from PubMed (n = 7; 50%), with European (n = 7; 50%) and American (n = 5; 35.75%) studies being the most common.

### 3.4. Synthesis Results

Study synthesis was conducted according to primary objectives and study design (clinical trials, observational studies, or reviews). Likewise, when relevant, sample size was considered to contextualize findings. Overall, studies highlight the emerging role of AI as a promising tool for early detection, prediction, and diagnosis of cognitive impairment. Most studies compared AI-based approaches with traditional methods and reported equal or superior performance. Additionally, several addressed the ethical implications of applying these technologies to vulnerable populations. Detailed characteristics, objectives, and conclusions are presented in Table 3, Table 4 and Table 5.

## 4. Discussion

In recent years, AI has become increasingly relevant in healthcare, particularly for the early detection of conditions such as cognitive impairment. This scoping review aimed to map the impact of AI-based tools for early detection. For this purpose, it has been essential to identify these tools and explore their effectiveness and ethical implications.

While previous reviews have examined the use of AI for dementia detection or cognitive decline prediction, many focus primarily on specific algorithms, single technological approaches, or particular biomarkers. In contrast, the present scoping review provides a broader mapping of AI-based tools for early detection of cognitive impairment, integrating evidence from diverse technological approaches, including imaging analysis, speech and gait assessment, electronic health records, and mobile health applications. Additionally, this review includes a discussion of ethical considerations and potential clinical applicability, aspects that have been less explored in many previous reviews.

### 4.1. AI-Based Tools for Early Detection of Cognitive Impairment

Early detection of cognitive impairment is essential to prevent progression to dementia, an area in which nursing plays a key role through continuous observation of patients’ cognitive status, enabling early identification of changes in higher functions such as memory, orientation, and behavior [24].

Traditionally, cognitive tests and brain imaging have been used; however, these methods are not always able to detect the early stages of cognitive decline due to the instability and gradual progression of its initial manifestations, as well as their limited sensitivity. Given the rapid expansion of AI in healthcare, its integration into early detection has been increasingly explored. The reviewed literature indicates that AI is becoming an important support tool for clinical decision-making through machine learning, deep learning, and natural language processing, which enable autonomous learning and performance improvement over time [24,26,27,33].

Based on this premise, proposed alternatives include the “Integrated Cognitive Assessment”, focused on rapid categorization tasks to measure patients’ processing speed, memory, and decision-making capacity, analyzed by AI to identify potential cognitive alterations [34].

Likewise, artificial neural network models combining cognitive, functional, and psychological tests have also been implemented. Supported by deep-learning techniques, these models allow a more comprehensive and accurate assessment than conventional tests alone [35].

Additionally, from a neuro-ophthalmological perspective, the retina is considered an extension of the central nervous system due to its structural and functional similarities. This close relationship allows neurodegenerative changes to manifest early at the retinal level. In this context, Shi et al. [33] showed that fundus photography, optical coherence tomography, and cognitive tests processed using deep learning can identify irregular ocular patterns acting as non-invasive biomarkers of cognitive impairment.

Furthermore, among the most accessible digital solutions, mobile applications, such as Cognity and Mobi-Cog, stand out. They use machine learning to analyze clock-drawing test performance and rapidly, non-invasively estimate the likelihood of cognitive impairment [25].

From another perspective, experimental methods based on automated gait and speech analysis are also being explored, allowing objective assessment of movement patterns and acoustic features from video and audio recordings. Alterations such as gait slowing, reduced verbal fluency, and prosodic changes may serve as early indicators of cognitive impairment, even in subclinical stages [24,26,27].

These tools are accessible, non-invasive, and applicable by nursing staff, facilitating integration into routine practice. Nevertheless, AI should be viewed as support for clinical decision-making rather than a replacement for professional clinical expertise, which is essential for tailoring the results to each patient’s individual situation and ensuring appropriate and safe care [26]. Notably, most of the studies included in this review focus on the development and validation of technological approaches for early detection of cognitive impairment rather than on their direct implementation in nursing practice. Consequently, evidence regarding the integration of AI tools into nursing workflows and their acceptability among nursing professionals remains limited.

### 4.2. Effectiveness of AI-Based Tools for Early Detection of Cognitive Impairment

According to this review, AI is consolidating as an effective support tool for early detection, not only because it can identify subtle patterns at early stages and process large datasets rapidly and accurately. Several studies reported improvements in the sensitivity and specificity of traditional cognitive screening approaches when artificial intelligence–based methods were applied, suggesting their potential to complement conventional assessment strategies [27].

In this context, studies evaluating the “Integrated Cognitive Assessment” conclude that this AI technology enhances healthcare system efficiency by reducing unnecessary referrals through faster and more accurate detection [34].

Furthermore, artificial neural networks, when combined with classical cognitive tests, have shown superior effectiveness compared with the use of individual tests [35].

Likewise, deep learning applied to retinal image analysis enables precise identification of cognitive impairment-related alterations, such as changes in retinal ganglion cells, arterial stenosis, and macular thickness variations associated with cerebral atrophy, and, consequently, related to cognitive impairment [33].

Some mobile applications have reported very high accuracy levels in detecting cognitive impairment, highlighting their potential as accessible screening tools. However, their diagnostic capacity remains limited regarding the cognitive domains assessed, such as complex attention, learning and memory, language, social cognition, executive functions, and motor perception [25].

Finally, gait and speech analysis using deep-learning techniques also show promising results as non-invasive biomarkers of cognitive decline. However, their performance may be affected by external interference and contextual variability. Specifically, deep-learning approaches applied to speech analysis have achieved high accuracy in detecting early cognitive changes, highlighting their potential as a non-invasive tool for early detection of cognitive impairment [24].

However, the performance of AI models reported in the included studies varies considerably across evaluation metrics and methodological approaches. Future research should report standardized performance indicators, such as sensitivity, specificity, and the area under the curve (AUC), to facilitate comparisons across AI models and improve the interpretability of results.

### 4.3. Ethical Implications of AI-Based Tools for Early Detection of Cognitive Impairment

The ethical implications of AI in early detection of cognitive decline include weighing the benefits against the risks for patients, caregivers, and healthcare professionals [26,27]. As this technology is still in the expansion and development phase, there is a lack of studies in the literature that thoroughly address these implications, leaving a knowledge gap for future research to explore. Nevertheless, from the nursing perspective, it is essential to consider the impact of these technologies on the quality of patient care [31].

The use of AI in individuals with cognitive disabilities raises ethical dilemmas, primarily related to clinical practice, as these systems collect confidential patient information, which may affect privacy and security. Ory et al. [36] note that its implementation can influence patients at a biopsychosocial level, an area where nursing has a significant impact.

Among the main benefits are improvements in the quality of patient care and the relevance of AI as a clinical decision-support tool, given its ability to analyze large-scale data quickly, identify patterns, and provide recommendations based on the most recent evidence. Nevertheless, its use also raises concerns regarding the potential replacement of human interaction, as well as emotional and social consequences, such as loss of trust, misuse of data, unemployment, or addiction [26,27,36].

This is partly due to the absence of specific legislation regulating these aspects and ensuring both the protection of sensitive information and respect for human rights in the use of AI technologies applied to neurodegenerative disorders [36]. This issue has been described by authors such as Depp et al. [26] and Alty et al. [27] as the “black box of machine learning,” referring to the lack of transparency and explainability of these systems.

Therefore, it is necessary to establish a specific ethical code of conduct for the use of AI in cognitive decline, as well as to ensure clear, transparent ethical governance to guide its development, validation, and clinical implementation [36].

Ethical considerations related to the implementation of AI should also be carefully addressed. Issues such as algorithmic bias, transparency and explainability of AI models, and the need to ensure informed consent when using patient data represent important challenges that must be considered when integrating these technologies into clinical practice [37,38].

### 4.4. Limitations

The main limitation of this review was the heterogeneity of the identified studies, combined with the relatively limited number of articles specifically addressing AI for the early detection of cognitive impairment. This heterogeneity in study design, methodologies, and technologies may have introduced variability that could influence the interpretation of the findings. Furthermore, restricting studies to those with freely accessible full text may have introduced an accessibility bias. Therefore, the conclusions of this review should be interpreted within the context of the open-access literature. Additionally, the search strategy was limited to peer-reviewed scientific literature. It did not include gray literature sources (e.g., reports, unpublished studies, or institutional documents), which may have limited the identification of additional evidence.

Moreover, during the search process, it was necessary to reformulate some of the initially proposed objectives due to the limited availability of suitable studies and the absence of prospective research on this topic.

Finally, although this review focused on recent literature published between 2020 and 2025 to capture the most current developments in AI applied to cognitive impairment detection, the rapid evolution of this field means that newly published studies may further expand the available evidence in the near future. Additionally, the inclusion of studies published only in English and Spanish may have excluded potentially relevant research published in other languages.

### 4.5. Future Research Directions

The relevance of this scoping review lies in identifying tools that facilitate nursing professionals’ early detection of cognitive decline, as they are the group most frequently detecting this condition due to closer patient contact, positioning them in a key role in recognizing early signs. In light of the results, there is a clear lack of current research on this approach, highlighting the need to promote new studies aimed at developing more adaptive and accurate algorithms to enhance diagnostic capacity in the context of cognitive decline.

Furthermore, the findings of this review support the utility of AI in this context, suggesting that its application will increase progressively among healthcare and social welfare providers, thereby contributing to earlier, more efficient, and more personalized care. Given the rapid development of AI technologies, the body of evidence in this field is expected to grow substantially in the coming years. Therefore, future reviews may expand the search period to incorporate newly published studies and provide an updated overview of emerging tools and approaches for the early detection of cognitive impairment.

## 5. Conclusions

Based on the results, there is evidence of simple, sensitive, low-cost, non-invasive tools that can be easily applied by nursing staff, powered by AI, and focused on early detection of patients with cognitive decline. These include the “Integrated Cognitive Assessment,” the use of artificial neural networks combining cognitive, functional, and psychological tests, the application of deep learning with ocular imaging, mobile applications, and gait and speech analysis.

Likewise, the effectiveness reported for these tools suggests their potential for early and accurate screening, promoting adequate follow-up of patients with cognitive decline by improving sensitivity and specificity compared with traditional cognitive tests. Importantly, these technologies should be considered decision-support tools that assist healthcare professionals rather than autonomous systems capable of replacing clinical judgment.

Finally, the use of AI in the early detection of cognitive decline offers clinical benefits but also raises important ethical implications, particularly regarding privacy and security, which must be addressed in future studies.

## Figures and Tables

**Table 1:** Selection Criteria.

Inclusion Criteria	Exclusion Criteria
- Publications published between 2020 and 2025. - Articles with free full-text access. - Articles written in English or Spanish. - Studies on the applicability of AI in patients with cognitive impairment. - Articles meeting medium-to-high methodological quality criteria.	Duplicate records. Studies not available in full text. Studies not related to the topic. Publications with low methodological quality.

**Table 2:** Search Strategies.

	Search Strategies
Search 1	((((((artificial intelligence) OR (machine learning)) OR (deep learning)) OR (artificial intelligence[MeSH Terms])) OR (machine learning[MeSH Terms])) AND (((premature detection) OR (early detection)) OR (diagnosis[MeSH Terms]))) AND ((((cognitive impairment) OR (cognitive dysfunction)) OR (neurocognitive impairment)) OR (cognitive dysfunction[MeSH Terms]))
Search 2	((((((((artificial intelligence) OR (machine learning)) OR (deep learning)) OR (artificial intelligence[MeSH Terms])) OR (machine learning[MeSH Terms])) AND (((premature detection) OR (early detection)) OR (diagnosis[MeSH Terms]))) AND ((((cognitive impairment) OR (cognitive dysfunction)) OR (neurocognitive impairment)) OR (cognitive dysfunction[MeSH Terms]))) AND (((((((hospitalization) OR (primary care)) OR (health center)) OR (healthcare settings)) OR (hospitalization[MeSH Terms])) OR (primary health care[MeSH Terms])) OR (community health centers[MeSH Terms]))) AND ((((viability) OR (feasibility)) OR (implementation)) OR (feasibility study[MeSH Terms]))
Search 3	((((((artificial intelligence) OR (machine learning)) OR (deep learning)) OR (artificial intelligence[MeSH Terms])) OR (machine learning[MeSH Terms])) AND ((((cognitive impairment) OR (cognitive dysfunction)) OR (neurocognitive impairment)) OR (cognitive dysfunction[MeSH Terms]))) AND ((ethic*) OR (ethics committee[MeSH Terms]))

**Table 3:** Identification Characteristics of the Selected Articles.

No.	Title	Authors/Year	Country
1	Revolutionizing Early Alzheimer’s Disease and Mild Cognitive Impairment Diagnosis: A Deep Learning MRI Meta-Analysis	Han, C.G. et al.; 2024 [23]	Brazil
2	Machine Learning Approaches for Dementia Detection through Speech and Gait Analysis: A Systematic Literature Review	Åberg, A.C. et al.; 2024 [24]	Sweden
3	Dementia Medical Screening Using Mobile Applications: A Systematic Review with a New Mapping Model	Hathurusingha, C. et al.; 2020 [25]	United Kingdom
4	Artificial Intelligence Approaches to Predicting and Detecting Cognitive Decline in Older Adults: A Conceptual Review	Depp, C.A. et al.; 2020 [26]	United States
5	Applications of Artificial Intelligence to Aid Early Detection of Dementia: A Scoping Review on Current Capabilities and Future Directions	Alty, J. et al.; 2022 [27]	United Kingdom
6	Digital Biomarkers for the Early Detection of Mild Cognitive Impairment: Artificial Intelligence Meets Virtual Reality	Cavedoni, S. et al.; 2020 [28]	Italy
7	Development and Validation of a Deep Learning Model for Earlier Detection of Cognitive Decline from Clinical Notes in Electronic Health Records	Amariglio, R.E. et al.; 2021 [29]	United States
8	Prediction of Decline in Global Cognitive Function Using Machine Learning with Feature Ranking of Gait and Physical Fitness Outcomes in Older Adults	Noh, B. et al.; 2021 [30]	South Korea
9	Use of Artificial Intelligence Techniques for Detection of Mild Cognitive Impairment: A Systematic Scoping Review	Heikkonen, M.R. et al.; 2023 [31]	Finland
10	Machine Learning Algorithms Based on Screening Tests for Mild Cognitive Impairment	Park, J.H.; 2020 [32]	United States
11	Deep Learning Models for the Screening of Cognitive Impairment Using Multimodal Fundus Images	Shi, X.H. et al.; 2024 [33]	China
12	The Use of a Computerized Cognitive Assessment to Improve the Efficiency of Primary Care Referrals to Memory Services: Protocol for the Accelerating Dementia Pathway Technologies (ADePT) Study	Apostolou, P. et al.; 2022 [34]	United Kingdom
13	A Real-Time Clinical Decision Support System for Mild Cognitive Impairment Detection, Based on a Hybrid Neural Architecture	Cabrera-León, Y. et al.; 2021 [35]	Spain
14	Emerging Issues of Intelligent Assistive Technology Use Among People with Dementia and Their Caregivers: A U.S. Perspective	Ory, M.G. et al.; 2020 [36]	United States

**Table 4:** Methodological Characteristics of the Selected Articles.

No.	Study Duration	Database	Included Studies/Patients
1	Systematic Review with Meta-Analysis	PubMed	18 studies.
2	Systematic Review	PubMed	40 studies.
3	Systematic Review	PubMed	20 mobile applications.
4	Conceptual Review	Scopus	21 studies.
5	Scoping Review	Scopus	177 studies.
6	Literature Review	Scopus	27 studies.
7	Diagnostic Study	Scopus	Clinical notes from 2166 patients.
8	Qualitative Study	Scopus	306 patients aged 75 or older.
9	Scoping Review	Scopus	70 studies.
10	Diagnostic Study	Cochrane	103 healthy patients and 74 with cognitive impairment.
11	Cross-Sectional Study	PubMed	9424 retinal photographs and 4712 optical coherence tomography images.
12	Study Protocol	PubMed	86 patients.
13	Diagnostic Study	PubMed	203 healthy patients and 128 with cognitive impairment.
14	Narrative Review	PubMed	27 studies.

**Table 5:** Objectives and Main Findings of the Selected Articles.

No.	Objectives	Conclusions
1	To analyze the diagnostic accuracy of deep learning applied to magnetic resonance imaging for the diagnosis of Alzheimer’s disease and cognitive impairment.	Deep-learning models based on magnetic resonance imaging provide a personalized, non-invasive tool for early detection of Alzheimer’s disease and cognitive impairment, improving diagnostic accuracy through high sensitivity and specificity. Early detection enables the implementation of more effective treatment strategies.
2	To summarize the existing literature on machine-learning models focused on gait and speech, using non-invasive methods, for the prediction of dementia.	Automated analysis of speech and gait using non-invasive machine-learning algorithms has demonstrated substantially higher accuracy in dementia prediction than manual analysis. Improvements in the performance of these machine-learning models are achieved by combining linguistic, acoustic, and cognitive features for speech analysis with motor features of gait.
3	To identify the availability of mobile applications for the detection of dementia and cognitive impairment by analyzing them in terms of comprehensiveness, validity, performance, and adaptation of AI and machine-learning techniques.	Screening applications for neurocognitive disorders, such as ALZ and CognitiveExams, are available for detecting dementia. Other applications incorporate AI and machine-learning techniques to improve the accuracy and efficiency of screening, such as Cognity and ACE-Mobile. In this study, applications were mapped by linking medical tests to the cognitive domains they cover, providing information on which dementia detection applications monitor a greater number of cognitive domains. Most of these applications can be used in both primary care and clinical settings and support patients, caregivers, families, and healthcare professionals in the detection of dementia and cognitive impairment.
4	To describe the uses, benefits, and limitations of artificial intelligence algorithms for predicting, diagnosing, and classifying cognitive impairment.	AI has the potential to transform the diagnosis and treatment of patients with neurocognitive disorders. Given the wide range of biopsychosocial characteristics of each individual, an individualized approach is required to improve the performance and clinical utility of machine-learning algorithms for predicting, detecting, and diagnosing cognitive impairment.
5	To use AI–assisted digital biomarkers for the early detection of dementia.	Early dementia screening tests show improved performance when they incorporate AI-based digital biomarkers, which can collect more relevant data in a single assessment than traditional methods. In addition, they provide more reliable data by eliminating subjective human components, thereby improving specificity and sensitivity. Speech, conversation, and language tests, as well as movement-based assessments, improve their diagnostic accuracy when combined with deep-learning techniques.
6	To develop a predictive model of cognitive impairment through the identification of patterns of cognitive and motor decline.	A cognitive–behavioral assessment of neuronal alterations and cognitive decline in older adults would allow the detection of cognitive impairment and dementia at early stages, thereby improving their management. This review integrates virtual reality, gait analysis, and machine learning to enable early detection and prediction of cognitive impairment. Virtual reality allows the collection of digital biomarkers directly related to brain function. Applying machine learning to these digital biomarkers enables the identification of cognitive–behavioral patterns and the development of a predictive model of the potential progression of cognitive impairment.
7	To develop a deep-learning-based model to detect cognitive impairment using electronic health records.	A deep-learning model trained on clinical notes from electronic health records has accurately detected cognitive impairment in patients prior to diagnosis. Using electronic health record data, the AI–based model incorporates prior diagnostic codes, medications, and patients’ problem lists.
8	To identify salient gait and physical fitness characteristics using machine-learning models to predict cognitive impairment in older adults.	A machine-learning-based approach enabled the identification of relevant gait and physical fitness features in a specific group of older adults to predict potential cognitive decline.
9	To explore different types of AI models for the detection of cognitive impairment.	The use of AI to detect cognitive impairment and to predict its progression to Alzheimer’s disease may represent a crucial advance in contemporary healthcare, as early detection facilitates a more favorable disease course for patients.
10	To compare the clinical effectiveness of machine-learning algorithms with traditional tests for the early detection of cognitive impairment.	Machine-learning algorithms designed to predict cognitive impairment were shown to be more effective than conventional detection techniques. In addition, these methods demonstrated the ability to rapidly differentiate between healthy individuals and patients with amnestic cognitive impairment.
11	To develop a deep-learning model based on ocular imaging to differentiate healthy individuals from those with cognitive impairment.	Examinations that can be readily performed in community-based settings or in clinical settings, such as fundus examination and optical coherence tomography, provide essential information on cognitive function, particularly cognitive impairment. These methods should therefore be considered in the detection of cognitive impairment in patients in primary care or inpatient settings.
12	To develop integrated cognitive assessment tools for use as cognitive impairment screening, thereby improving dementia care.	ADePT aims to improve the care of patients with dementia in both primary and specialized care by supporting appropriate decision-making regarding diagnosis and treatment and reducing healthcare system burden due to unnecessary referrals.
13	To explore the utility of artificial neural networks to facilitate reliable detection of cognitive impairment and to compare these techniques with traditional clinical screening tools.	An artificial neural network system based on cognitive and functional domains was designed to detect cognitive impairment in primary care and outpatient neurology and geriatrics settings, considering age and educational level as modifying factors, the latter being less relevant.
14	To explore barriers to access to AI–based intelligent assistive technologies for patients and caregivers, as well as the ethical, legal, and social implications of AI design and implementation in dementia care.	Access to intelligent assistive technologies for patients with cognitive impairment and caregivers should be affordable, ethical, easy to use for vulnerable populations, and free of challenges related to socioeconomic status or technological literacy. These technologies may be beneficial by promoting independence, safety, and protection for people with dementia; however, they may also pose risks to rights, privacy, dignity, and autonomy. Therefore, from an ethical perspective, safeguarding human rights and equity for patients is essential.

## Data Availability

No new data were created or analyzed in this study. Data sharing is not applicable to this article.

## Abbreviations

The following abbreviations are used in this manuscript:

ADePT	Accelerating Dementia Pathway Technologies
AI	Artificial Intelligence
CASPe	Critical Appraisal Skills Programme in Spanish
JBI	Joanna Briggs Institute
MeSH	Medical Subject Headings
MMAT	Mixed Methods Appraisal Tool
PRISMA-ScR	PRISMA Extension for Scoping Reviews
